# Bacteria as Biological Control Agents of Plant Diseases

**DOI:** 10.3390/microorganisms10091759

**Published:** 2022-08-31

**Authors:** Anna Bonaterra, Esther Badosa, Núria Daranas, Jesús Francés, Gemma Roselló, Emilio Montesinos

**Affiliations:** Laboratory of Plant Pathology, Institute of Food and Agricultural Technology-CIDSAV-Xarta, University of Girona, Campus Montilivi, 17071 Girona, Spain

**Keywords:** bacterial biological control agents, bacterial and fungal plant diseases, screening, improvement

## Abstract

Biological control is an effective and sustainable alternative or complement to conventional pesticides for fungal and bacterial plant disease management. Some of the most intensively studied biological control agents are bacteria that can use multiple mechanisms implicated in the limitation of plant disease development, and several bacterial-based products have been already registered and marketed as biopesticides. However, efforts are still required to increase the commercially available microbial biopesticides. The inconsistency in the performance of bacterial biocontrol agents in the biological control has limited their extensive use in commercial agriculture. Pathosystem factors and environmental conditions have been shown to be key factors involved in the final levels of disease control achieved by bacteria. Several biotic and abiotic factors can influence the performance of the biocontrol agents, affecting their mechanisms of action or the multitrophic interaction between the plant, the pathogen, and the bacteria. This review shows some relevant examples of known bacterial biocontrol agents, with especial emphasis on research carried out by Spanish groups. In addition, the importance of the screening process and of the key steps in the development of bacterial biocontrol agents is highlighted. Besides, some improvement approaches and future trends are considered.

## 1. Introduction

Plant pathogens constitute a great threat to agricultural and forestry production since they cause diseases with important economic and environmental impact [1,2]. Currently, their effect has worsened due to globalization of markets and global climate change that facilitate the appearance of emerging diseases and their rapid spread [3]. New trends in crop protection have been oriented toward a reduction of reliance on conventional pesticides together with the compulsory implementation of integrated pest management (IPM) principles program addressed in the regulations of different countries [4,5]. Consequently, the interest in effective and sustainable alternative strategies to conventional pesticides has increased. Biological control is regarded as a promising alternative and a wide array of microbial biocontrol agents (BCA) have been developed in the past decades for the management of fungal and bacterial diseases. Some of the most intensively studied are bacteria belonging of the genus *Pseudomonas* spp., *Bacillus* spp., and *Streptomyces* spp., that have been already registered as commercial products and marketed. Nowadays, in EU there are 13 bacterial-based biocontrol agents (BCA) registered as biopesticides for the control of bacterial and fungal diseases (*Bacillus amyloliquefaciens* strains: QST 713, AH2, MBI 600, FZB24 and IT 45, *Bacillus amyloliquefaciens* subsp. *plantarum* strain D747, *Bacillus firmus* I-1582, *Bacillus pumilus* strain QST 2808, *Bacillus subtilis* strain IAB/BS03, *Pseudomonas* sp. strain DSMZ 13134, *Pseudomonas chlororaphis* strain MA 342, *Streptomyces* K61 and *Streptomyces lydicus* strain WYEC 108) (https://food.ec.europa.eu/plants/pesticides/eu-pesticides-database_en, accessed on 1 June 2022). However, efforts are still required to increase the commercially available microbial biopesticides for plant disease management [6].

The efficacy of a bacterial biocontrol agent against plant diseases depends on the microbial agent (mechanism of action, conditioning, dose, methods of application), plant pathogens targets (sensitivity), host (cultivar type, physical properties), and environmental conditions (biotic and abiotic factors, chemical residues, nutrient availability, temperature, moisture) [7]. Numerous interactions may affect the efficacy of biocontrol such as the variability from plant to plant, orchard, and year, and often lack of efficacy and inconsistent field performance have been reported. Therefore, it is necessary to know the efficacy and consistency of biological control in comparison to standard chemical fungicide and bactericide treatments under sufficiently wide production conditions in orchards representing different environments and agricultural practices [8,9].

**Figure 1:**
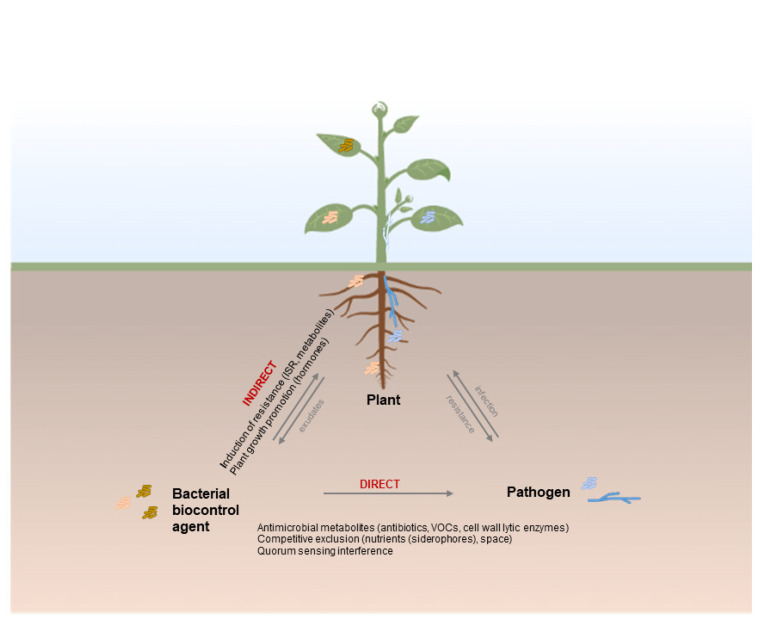
Overview of the direct and indirect mechanisms of biocontrol involving interaction between bacterial biocontrol agent, pathogen, and plant (created with BioRender.com).

Bacterial biocontrol agents use a great variety of mechanisms to protect plants from pathogen infections. They may use one or a combination of mechanisms to prevent or reduce plant disease, interacting directly or indirectly with the pathogen [10,11] (Figure 1). BCA can interact directly with the pathogen through the secretion of antimicrobial compounds, interfering with the pathogen virulence and competing for nutrients and space. Many BCA synthesize and release metabolites such as lipopeptides, bacteriocins, antibiotics, biosurfactants, cell-wall degrading enzymes or microbial volatile compounds which have antimicrobial activity by reducing growth or metabolic activity of pathogens. BCA may also interfere with the quorum sensing (QS) system of the pathogens, enzymatically degrading or inhibiting the synthesis of signal molecules used to initiate infections. For instance, producing QS inhibitors such as lactonases, pectinases, and chitinases that degrade QS signal molecules impairing pathogen infection and reducing the symptoms of plant diseases [12]. Moreover, BCA can diminish pathogen infection pressure through competitive exclusion over pathogens by reducing their growth without killing them. Highly competitive bacterial BCA may colonize and survive in the infection site and have a more efficient nutrient uptake system than the pathogen, such as low-molecular-weight siderophores with affinity for ferric iron. Besides direct interactions, BCA can protect plants indirectly, by triggering the defense response or promoting plant growth [10,11,13]. They may enhance host defense mechanism eliciting systemic resistance. This results in an accumulation of structural barriers and triggers many biochemical and molecular defense responses in the host, conferring a protective system against a wide range of pathogens. Moreover, BCA can promote plant growth by enhancing mineral and water absorption or producing plant growth stimulating compounds, such as hormones, and thereby improving plant health and fitness. In many cases, various mechanisms are involved in the complex interactions between plants, BCA, and pathogens. Therefore, identifying the mechanisms responsible for biocontrol is a great challenge. Understanding the mode of action responsible for the protective effect of a BCA will facilitate the optimization of biocontrol and allow the establishment of optimal conditions for the interaction between the BCA, the pathogen, and the host, and the design of appropriate formulations and methods of application to enhance plant health and sustainable agriculture.

This review shows some relevant examples of known bacterial BCA, and presents their main modes of action, including details concerning the mechanisms and molecules involved in the biocontrol activity with especial emphasis on research carried out by Spanish groups. In addition, the importance of the isolation, screening process, characterization of the key steps in the development of BCA is highlighted. Moreover, some improvement approaches and future trends are considered.

## 2. Bacteria as Biological Control Agents of Plant Diseases

A wide variety of bacterial genera, including Agrobacterium, Alcaligenes, Arthrobacter, Bacillus, Enterobacter, Erwinia, Pseudomonas, Rhizobium, Serratia, Stenotrophomonas, Streptomyces, and Xanthomonas have been described to have plant disease protection activity against fungal and bacterial pathogens. These bacteria can use multiple mechanisms implicated in the limitation of plant pathogens development. These mechanisms of action include colonization of infection sites and competitive exclusion of the pathogen, antagonistic activity based on the secretion of highly active antimicrobials such as antibiotics or cell wall lytic enzymes and induction of plant resistance [7,14,15].

Several bacterial BCA of bacterial and fungal pathogens have been developed in research carried out within the framework of Spanish groups and some examples are highlighted (Table 1).

### 2.1. Pseudomonas *spp.*

Fluorescent pseudomonads are ubiquitously present in plant environments and possess several relevant traits for their effectiveness in the reduction of plant diseases. These traits include a high ecological fitness, a strong antagonistic activity toward various plant pathogens, and a potent ability to trigger an immune reaction in plant.

Many *Pseudomonas* spp. are efficient colonizers of the plant surface (rhizosphere and phyllosphere) and the endosphere. They can use many plant exudates as nutrients and have a high growth rate, which are prerequisites to efficiently compete with other microorganisms for space and nutrients in the plant environment [37,38,39]. For example, the activity of *P. fluorescens* EPS62e and *P. pseudoalcaligenes* AVO110 in the reduction of *Erwinia amylovora* or *Rosellinia necatrix* infections, respectively, is based on their strong fitness in colonizing plant tissues as they have higher growth potential and nutrient use efficiency than the target pathogens [29,33]. In addition, competition for limited nutrients has been described as an important mechanism of *Pseudomonas* spp., but it is only relevant when the concentration of a given limited nutrients is low, such as in the biological control of *Pythium ultimum* by *P. fluorescens* 54/96 [40] or in the case of siderophore-mediated competition for iron in the reduction of *Fusarium* wilt of carnation by *P. putida* WCS358 [41].

Another relevant trait of *Pseudomonas* spp. is that they are major producers of bioactive metabolites, such as antibiotics, cyclic peptides, or enzymes that play important ecological roles. Specifically, they produce different antimicrobial compounds such as phenazines, phloroglucinols, dialkylresorcinols, pyoluteorin, and pyrrolnitrin, whose involvement as a mechanism of action in biological control has been well documented [38,42]. Phenazines such as phenazine-1-carboxamide (PCN) or phenazine-1-carboxylate (PCA) are nitrogen-containing heterocyclic compounds with broad antifungal and antibacterial activities. These compounds are involved in the reduction of fungal pathogens infections of plants. For example, PCN produced by *P. chlororaphis* subsp. *aurantiaca* strain Pcho10 shows strong inhibitory activity against *Fusarium graminearum* [43] and PCA produced by *P. fluorescens* EPS894 inhibits *Phytophthora cactorum* in strawberry plants [30]. The phloroglucinols are phenolic broad-spectrum antibiotics produced by a wide variety of bacterial strains. Specifically, 2,4-diacetyl phloroglucinol (DAPG), produced by different strains of *Pseudomonas* spp., has a broad-spectrum action, and contributes to the biological control of plant disease, especially soil-borne plant diseases [28,44]. Dialkylresorcinols exhibit antifungal and antibacterial activities such as the compound 2-hexyl-5-propyl resorcinol produced by *P. chlororaphis* PCL 1606 is responsible for the biocontrol of *R. necatrix* [27]. Pyrrolnitrin have also been involved in the biocontrol of the *Fusarium* head blight by *P. chlororaphis* G05 [45]. Pyoluteorin, as well as the volatile compound hydrogen cyanide are other compounds produced by different strains of *Pseudomonas* spp. that have been involved in the biocontrol of some pathogens [46].

Moreover, pseudomonads produce cyclic lipopeptides (CLPs) that are amphiphilic molecules containing chains of 7–25 aminoacids of which several form a lactone ring coupled to a fatty acid tail. Many of the CLPs are biosurfactants, which can damage cell membranes, thereby causing leakage and cytolysis and are a common feature of both plant beneficial and pathogenic bacteria [46,47]. Interestingly, some of them such as orfamides synthesized by *P. protegens* have antimicrobial activity against a variety of organisms, including the pathogenic oomycetes *Pythium* and *Phytophthora*, and the fungus *Rhizoctonia* [48]. Other examples that show antifungal activity are the cyclic depsipeptide viscosinamide produced by *P. fluorescens* DR54 [49] or the peptide tensin produced by *P. fluorescens* 96.578 [50].

Pseudomonads can also produce lytic extracellular enzymes such as chitinases, β-1,3 glucanases, cellulases that have important roles in biocontrol activity by their degradative activities of cell wall compounds, such as chitin, glucan, and glucosidic bridges. For example, hydrolytic enzymes produced by *Pseudomonas* sp. have in vitro antifungal activity against *Pythium aphanidermatum* and *Rhizoctonia solani* and promote growth in chickpea [51].

*Pseudomonas* spp., can trigger defense responses of host plants through different pathways, conferring plants with resistance to multiple pathogens. In many cases they confer resistance to plant upon the activation of induced systemic resistance (ISR) that involves activation of immune response and priming state for a more efficient activation of defenses. For example, in *Vitis*, *P. fluorescens* PTA-CT2 induces ISR to *Plasmopara viticola* and *Botrytis cinerea* that depends on the activation of SA or JA and ABA defensive pathways [52]. In another case, the biocontrol endophytic bacterium *Pseudomonas simiae* PICF7 induces systemic defense responses in aerial tissues upon colonization of olive roots [31,32]. In addition, some compounds such as CLPs or phenazines have been reported to trigger defense responses in plants. For example, massetolide A of *P. fluorescens* enhanced resistance to infection by *Phytophthora infestans* in tomato plants [53] and phenazines from *Pseudomonas* sp. CMR12a induced systemic resistance on rice and bean [54].

### 2.2. Bacillus *spp.*

*Bacillus* species are among the most exploited beneficial bacteria as biopesticides. They are widely distributed in several habitats such as soil and plant surfaces, have broad physiological ability and capability to form endospores that confers resistance to adverse environmental conditions. They can develop antagonism against a wide range of bacterial and fungal plant pathogens. The most remarkable trait of *Bacillus* spp. is the ability to produce a wide variety of bioactive compounds valuable for agricultural applications, including metabolites with antimicrobial activity, surface-active, and implicated in the induction of plant defense responses [55,56].

Bacteriocins and bacteriocin-like substances are ribosomally synthesized peptides that act against target cells by interfering with the synthesis of the cell wall or by forming pores in the cell membrane. *Bacillus* spp. produce several bacteriocins with antimicrobial activity such as amylolysin, amylocyclicin, amysin, subtilin, subtilosin A, subtilosin B, thuricin [57]. Some of them have been involved in biocontrol of plant pathogens. For example, Bac-GM17 produced by *B. clausii* GM17 have activity against *Agrobacterium tumefaciens* [58] or thuricin Bn1 from *B. thuringiensis* subsp. *kurstaki* Bn1 against *Pseudomonas savastanoi* and *Pseudomonas syringae* [59].

Cyclic lipopeptides (CLPs) are non-ribosomally synthetized amphiphilic compounds, composed of a fatty acid tail linked to a short oligopeptide which form a macrocyclic ring structure that are widely spread in *Bacillus* spp. The most important CLPs produced by *Bacillus* are represented by iturins, fengicins, and surfactins. They interact with cell membrane of target pathogens forming pores and leading to an imbalance in transmembrane ion fluxes [60]. There are several examples of *Bacillus* spp. strains producing CLPs, that are responsible for the antifungal activity that protect plants from diseases. The fengycin, iturin A, and surfactin produced by *B. amyloliquefaciens* PPCB004 and bacillomycin, fengycin, and iturin A produced by *B. subtilis* UMAF6614 and UMAF6639 are key factors in the antagonism against fungal pathogens [16,18]. In addition, *Bacillus* strains producing CLPs have also antibacterial activity such as *B. amyloliquefaciens* A17 (currently *B. velezensis*) that produces bacillomycin, fengycin, iturin, and surfactin which act synergistically against several bacterial plant pathogens [19,20], or *B. amyloliquefaciens* KPS46 that produces surfactin, required to reduce infections by *Xanthomonas axonopodis* pv. glycines [61]. In many cases, lipopeptides and other peptides or volatile organic compounds (VOCs) act in a synergistic manner to improve their activity. For example, *B. amyloliquefaciens* CPA-8 produces fengycin and VOCs that are involved in the antifungal activity against *Monilinia* and *Botrytis* [17]. Besides their antimicrobial activity, some of these compounds act indirectly as elicitors of defense mechanism in the host plant or play an important role in favoring colonization [62].

Hydrolytic enzymes such as chitinases, chitosanases, glucanases, cellulases, lipases, and proteases, are also extensively produced by *Bacillus* spp. strains. These compounds efficiently hydrolyze the major components of the fungal and bacterial cell walls and have been involved in plant pathogen suppression. For example, a protease produced by *B. amyloliquefaciens* SP1 showed efficacy in biocontrol of *Fusarium oxysporum* [63] and the hydrolase activity (protease, chitinase, cellulase, glucanase) was identified as the key factor of *B. velezensis* in controlling pepper gray mold caused by *Botrytis cinerea* [64].

Various *Bacillus* spp. strains can elicit ISR in different plants and confer an enhanced defense mechanism against a range of pathogens. Several studies have shown that VOCs and CLPs, such as surfactin and fengycin, are involved in the immune response of plants elicitation [65,66]. For example, *B. amyloliquefaciens* FZB42 produced secondary metabolites (surfactin, fengycin, and bacillomycin D) that trigger plant defense gene expression and contribute to lettuce bottom rot reduction [67]. In another example, *Bacillus subtilis* OTPB1 increased the levels of growth hormones and defense-related enzymes in tomato, conferring protection against early and late blight [68].

### 2.3. Other Relevant Bacteria as BCA

There are other relevant species/strains which can be used to develop microbial biopesticides. Some are distributed among the Gram-negative bacteria of the families Rhizobiaceae, Enterobacteriaceae, and Xanthomonadaceae. Others can be found among Gram-positive bacteria such as Lactobacillaceae, Leuconostocaceae, and Streptomycetaceae [69]. Some examples, since they reduce plant pathogenic bacteria and fungi infections, include species of *Streptomyces* spp., *Pantoea* spp., and *Lactobacillus* spp.

*Streptomyces* spp. is one of the most studied genus of bacteria, since they produce bioactive compounds that inhibit plant pathogens in vitro and are effective in the controlling various bacterial and fungal plant diseases [70]. Examples of such metabolites include macrolide benzoquinones, aminoglycosides, polyenes, and nucleosides. *Streptomyces* strains are also known for their ability to produce extracellular enzymes active in fungal cell wall degradation. These hydrolases may be responsible for the mycoparasitic potential of some strains and the limitation of plant diseases, such as in the strains *Streptomyces* CBQ-EA-2 and CBQ-B-8 that have chitinolytic, cellulolytic, and proteolytic activity and reduced *Macrophomina phaseolina* and *Rhizoctonia solani* infections in *Phaseolus vulgaris* [34]. Other bioactive metabolites are produced, including VOCs, as signaling molecules to regulate plant growth and immunity in response to biotic and abiotic stresses. In addition, some strains can limit plant disease development through the induction of systemic resistance (ISR) in plants. ISR elicited by *Streptomyces* strains occurs via the activation of the jasmonic acid/ethylene and salicylic acid pathways. For example, *S. lydicus* M01 treatment reduced the reactive oxygen species (ROS) accumulation and increased the activities of antioxidases related with ROS scavenging, which indicated an enhanced resistance of cucumbers against *Alternaria alternata* foliar disease [71]. Predominantly, these bacteria are obtained from the soil, and from the endosphere and rhizosphere of plants. As an example, *Streptomyces* sp. endophytic strain VV/E1 and rhizosphere VV/R1 and VV/R4 strains exhibited antifungal activity and reduced nursery fungal graft infections on grapevine plants [35].

Many strains of *Pantoea* spp. have aptitudes as BCA because they are ubiquitous and produce antimicrobial compounds. Biopesticides based on *Pantoea* spp. are registered and commercially available in Canada, USA, and New Zealand. They have biocontrol activity through various mechanisms, including competitive colonization, production of antimicrobials, and/or induction of host systemic defense. Some strains of *Pantoea* species have been shown to target a wide spectrum of plant pathogens including bacteria, fungi, and oomycetes via secretion of antimicrobial compounds such as pantocins, herbicolins, microcins, and phenazines [72,73]. Other strains such as *P. agglomerans* EPS125 or strain CPA-2 require direct cell-to-cell interaction to combat postharvest fungal pathogens, without relying on the production of antibiotic substances or nutrient competition [24,25,26]. In another example, *Pantoea* species can also produce N-acyl-homoserine lactone (AHL), affecting quorum sensing in pathogens which, coupled with promoting environmental fitness in plants, may contribute to limit pathogen development [74].

Lactic acid bacteria (LAB) are good candidates as BCA because they include some strains categorized as Generally Regarded as Safe (GRAS) by the U.S. Food and Drug Administration (FDA) and as having Qualified Presumption of Safety (QPS) status by European Food Safety Authority (EFSA) and have been widely reported as biopreservatives of vegetables and fruits [23]. LAB show antimicrobial activity due to the production of one or more antimicrobial metabolites. These include organic acids, carbon dioxide, diacetyl, hydroxide peroxide and proteinaceous compounds such as bacteriocins and antifungal peptides. They may also exclude pathogens by pre-emptively colonizing plant tissues susceptible to infection, by competition for nutrients and space, or by inducing defense responses in plants. For example, *L. plantarum* PM411 and TC92 are effective in preventing bacterial plant diseases. Their broad spectrum of antagonism against plant pathogenic bacteria is based on antimicrobial metabolites, together with the reduction of infections by inhibition of pathogen population on plant surfaces [21,22,75]. Moreover, *Weissella cibaria* TM128 exhibited antimicrobial activity and prevented blue mold, mainly due to the production of organic acids and hydrogen peroxide [36].

## 3. Bacterial Biocontrol Agent’s Development—Flowchart of Actions

**Figure 2:**
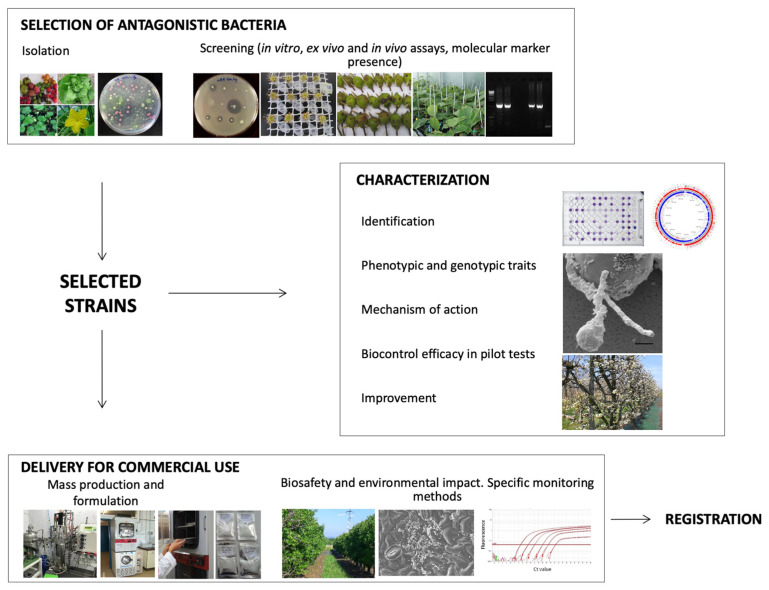
Flowchart of actions for bacterial biocontrol agents development.

The development of bacterial BCA requires several steps (Figure 2). It includes: (i) The isolation and selection of strains by means of screening methods able to analyze a high number of microorganisms; (ii) the characterization of the BCA, including the identification, the determination of phenotypic and genotypic traits, and the mechanisms of action, biocontrol efficacy in pilot tests and improvement; (iii) mass production and an appropriate formulation, which allow increasing biocontrol activity and ensuring its stability. Finally, the development of a monitoring system to detect and quantify the BCA in the environment and to make more extensive toxicology tests or environmental impact studies with the aim to register for use is required.

### 3.1. Isolation and Screening for Strain Selection

The first stage of BCA development consists of the isolation and screening of isolates able of limiting the development of the targeted plant pathogen and reducing disease levels. Proper sampling at adequate niches can increase the probability of obtaining useful strains, therefore careful selection of the origin of samples, culture media composition, and enrichment-isolation techniques is very decisive [8]. Bacterial antagonists that prevent or limit disease development are naturally present in the plant environment (phyllosphere, rhizosphere, and endosphere) or in bare soil. Different habitats can be used as suitable sources to obtain candidates as BCA.

For example, samples may be taken from suppressive soils or healthy plants from epidemic areas, where there is evidence of presence of beneficial microorganisms, or near the pathogen infection site [8,9]. In addition, other habitats different from the plant environment can also allow to obtain beneficial bacteria. As the presence of microorganisms with suitable properties as BCA is relatively rare in a strain collection, the isolation of a high number of candidates is recommended. The choice of the isolation technique using selective and enrichment culture media allows for the successful isolation of microorganisms of interest. However, this approach restricts the type of microorganisms obtained and few bacteria genera have been systematically evaluated as BCA. Another approach deals with the use of molecular markers to prospect BCA candidates by means of the specific detection of genes involved in the biocontrol and can be used as a good strategy to increase the efficiency of screening procedures [7,49,50]. The advances in genome sequencing and annotation, and the understanding of the mechanisms of action of BCA have greatly increased the availability of marker genes as tools for the screening [76]. Moreover, considering that a wide array of bacteria from different taxonomic group that studies the structure and function of plant microbiome have been identified [77,78], in-depth study of genetic diversity of microbial communities associated with plants can allow finding new bacteria with relevant traits related to biocontrol which can extend the candidates for plant diseases management [79].

Once a collection of isolates has been made, the putative BCA will be selected based on their attributes. The screening for appropriate candidates is a critical step in the development of novel bacterial BCA and determines the type of microorganism selected [7,9,80]. Rapid-throughput in vitro assays are widely used. In these assays, the target pathogen and candidate biocontrol agents are grown together in solid or liquid media to test for direct reduction of pathogen growth. These assays are fast, reproducible, and reliable, and allow the analysis of many isolates. However, they only permit the selection of bacteria with antagonistic activity, and they may not identify microorganisms with other mechanisms of action such as competitive exclusion or induction of plant resistance [10,81]. Screening procedures such as small-scale whole-plant bioassays in which pathogen and antagonists interact with the host in controlled conditions allow the selection of microorganisms with other mechanisms of action and have a good correlation with biocontrol efficacy in the field. However, these assays are time-consuming and require significant number of resources. The development of ex vivo bioassays on seeds, detached leaves, flowers, and fruits reduces plant material size and permits faster, reliable, and efficient screening [82,83]. A multi-pathogen approach is recommended to select strains with a broad spectrum of activity [74,84].

### 3.2. Characterization of Selected Strains

The deep characterization of the selected strains is an important stage of BCA development since it provides relevant information about strains for their exploitation as biopesticides. The identification, and phenotypic and genotypic characterization of the strains reveals key attributes in their activity as biocontrol agents. Some of these traits include the synthesis of compounds related to the antimicrobial activity such as enzymes, antibiotics, bacteriocins, or toxins that have detrimental activity against other microorganisms, or to their ability to trigger an immune reaction in plant tissues. Moreover, other traits contribute to the ability of a bacterial strain to colonize plant environment such as the efficient use and uptake of nutrients from exudates (amino acids, organic acids, sugars), motility (flagella), fast growth rate, ability to synthesize amino acids and vitamins, and presence of different structures for adhesion to plant surfaces, such as pili, fimbriae, major outer membrane proteins, or the O-antigen chain of lipopolysaccharides [85,86]. Understanding the traits that are involved as the mechanism of action of a BCA may help finding optimum conditions for implementing biocontrol in each pathosystem. However, the assessment of the mechanisms is a complex and difficult task because of the need of prospective studies to reveal the implication of a given process (e.g., antibiosis, nutrient competition, host colonization, induction of plant defense) and because, in most cases, there are several mechanisms involved and the importance of each one depends on the particular biotic and abiotic conditions.

Nowadays, the genome sequencing of BCA and its comparison with related published genomes will provide a framework for further functional studies of their colonization of plant environment competence and biocontrol effectiveness [87]. Comparative genomics between bacterial strains of varying biocontrol activities allow the identification of new candidate genes putatively involved in the biocontrol. This analysis will unravel novel insights into the biocontrol mechanisms of bacterial BCA and provide new resources for disease control [88,89].

Before bacterial strain is seriously considered for a microbial biopesticide development, pilot trials (greenhouse and field bioassays) must be conducted in several pathosystems and under diverse environmental conditions to ensure a wide range of applicability, as well as consistency in efficacy under real conditions [8]. Considering that the relative dose of pathogen and BCA is an important factor determining the efficacy and consistency of biological control, it is necessary to optimize the dose and frequency of applications. Dose–response models have been developed to obtain quantitative parameters that describe the efficacy of the BCA [90]. These parameters may give information on the dose range of the BCA needed to provide reliable, economical biological control, and allowing for the comparison of different BCA and pathosystems [8,69,90]. The required dose of BCA may be dependent on the mechanism by which a biocontrol agent performs its action. For a strain which acts via antibiosis or competitive exclusion it may be assumed that proper colonization is needed to deliver antimicrobial compounds or to compete with the pathogen, whereas for a strain which acts through ISR a smaller number of bacteria during a restricted period may be sufficient to elicit a successful response in the host plant [17].

### 3.3. Formulation and Delivery for Commercial Use

The final stages of B-BCA development include industrial scale production, formulation, and preservation. Suitable and cost-effective mass production at the industrial scale system must be carefully developed to obtain the highest number of cells in the shortest period. Moreover, it must be guaranteed that the production method does not alter the characteristics of the strains responsible for biocontrol. Culturing conditions determine population densities at the time of harvest and influence the viability and fitness of the microbes during formulation, storage, and application. These are however specific for each microbial strain and need to be screened carefully for improving final performance of microorganisms in the field [91]. Subsequently, developing an appropriate formulation (dry or liquid) is fundamental to increasing shelf-life, improving delivery, enhancing persistence in the field, and maintaining the viability and biocontrol efficacy [92]. Thus, the use of protective additives and adjuvants compatible with the BCA is common and they can be incorporated at different points of the production-formulation process. Classical protective substances (sucrose, glycerol, Arabic gum) improve survival of the microorganisms and adjuvants (surfactants, emulsifiers, dispersants, coupling agents, stabilizing agents) facilitate mixing, handling, application, and effectiveness [91].

In addition, biosafety studies must be undertaken to guarantee the lack of adverse effects of the active ingredient and the formulated product in plants and non-target organisms, including humans. It is also required to perform risk assessment studies on traceability, residue analysis, and environmental impact [8]. Thus, the development of reliable monitoring methods that accurately identify the released microorganism at strain level and track its population dynamics over time is a registration requirement [93]. Examples of strain specific quantitative monitoring methods developed for BCAs are real-time PCR for *P. fluorescens* EPS62e [94,95,96] or viable qPCR for *L. plantarum* PM411 [97]. These methods are useful for monitoring the fate and behavior of a released strain in the environment and for the quality control during production and formulation of the microbial biopesticide.

For placing the microbial biopesticide on the EU market, the active substance (i.e., bacterial BCA strain) needs to be approved at EU level and the formulated product must be authorized at Member State level (Regulation (EC) No 2009/1107 and (EC) 2017/1432). The registration procedure generally requires detailed dossiers accounting for scientific data on microorganism identity, biological properties, efficacy, specific analytical methods, residues, traceability, and potential adverse effects on human health and non-target organisms [8,93]. Microorganisms categorized as safe are highly appreciated for the development of microbial biopesticides. For example, bacteria designated with the GRAS and QPS status by the FDA and the EFSA, respectively, have a history of safe use in agriculture and in food and feed crops and lack known toxic or allergenic properties. These microorganisms are considered non-pathogenic to humans, or non-deleterious to the environment according. Therefore, the fact of belonging to this group facilitates the registration process for marketing.

## 4. Improvement of Biocontrol and Future Trends

The inconsistency in the performance of BCA in the biological control of phytopathogenic fungi and bacteria has limited their extensive use in commercial agriculture. Pathosystem factors such as host genotype, intrinsic characteristics of the pathogen, pathogen inoculum density, and environmental conditions have been shown to be key factors involved in the final levels of disease control achieved by bacteria. Multitude of biotic and abiotic factors can negatively influence the performance of the BCA, affecting their mechanisms of action or the multitrophic interaction between the plant, the pathogen, and the bacteria. However, some strategies can be adopted to improve the performance of BCA consisting of nutritional enhancement, physiological adaptation of BCA to stress and improvement of formulation (Table 2), as well as genetic manipulation of microorganisms. In addition, another challenge is to develop specific delivery systems that favor the success of biocontrol programs. Delivery methods must be carefully selected based on the characteristics of a particular BCA against a specific pathogen. Bacteria can be applied directly to seeds by different methods such as biopriming, encapsulation, or fluid drilling, to soil by drenching, mixing, or microbigation, and on plant aerial parts by foliar spraying or directly into the vascular system by means of endotherapy [98].

An improvement strategy of BCAs is based on nutritional enhancement, which consists of adding nutrients to the formulation that are more efficiently used by the biocontrol agent than by the pathogen. For example, the addition of glycine and Tween 80 to the formulation of *P. fluorescens* EPS62e improved its survival and adaptability in the plant environment [104] or the glucose analog, 2-deoxy-D-glucose enhanced biocontrol of blue mold on apples and pears [105]. Another effective approach to enhance the epiphytic establishment of BCA on plant surfaces is the physiological adaptation by osmoadaptation. This procedure based on the combination of saline osmotic stress and osmolyte amendment of the growth medium has been used to increase intracellular accumulation of osmolytes and drought stress tolerance. This strategy improved epiphytic survival and biocontrol efficacy of the apple blue mold biocontrol agent *P. agglomerans* EPS125 [101] and CPA-2 [106] and the fire blight biocontrol agents *P. fluorescens* EPS62e [102,103,104], *P. agglomerans* E325 [107] and *L. plantarum* PM411 [99].

The improvement of biocontrol can be achieved by application of mixtures of BCAs, the so-called consortia. This approach consists of designing mixtures of compatible strains that complement each other in terms of the mechanism of action and ecological attributes. This strategy may increase the efficacy and reliability of biocontrol in different environmental conditions, as well as provide a broader spectrum activity due to the synergistic effect of different mechanisms of action of the introduced biocontrol strains. Some examples are, dual mixtures of *P. fluorescens* and *Pantoea* sp. that enhanced the biocontrol of fire blight of pear [108], or mixtures of *P. fluorescens* producing different bioactive metabolites that improved the biocontrol of *P. cactorum* root rot in strawberry plants [30] and *P. infestans* in potato plants [109]. In some cases, the consortia include a high number of bacteria such in a consortium of seven different bacterial species used to protect maize against *Fusarium* [110] or a mixture of eight *Pseudomonas* strains that enhanced protection of tomato against bacterial wilt [111]. In addition, another possible strategy to improve the biocontrol efficacy is the amendment of BCAs with low toxic antimicrobial compounds. Several studies reported the combination with compounds such as bioregulators, organic acids, or essential oils. Improved biological control was reported by combining *L. plantarum* strains PM411 and TC92 with lactic acid [100], and *Bacillus amyloliquefaciens* or *L. plantarum* strains with essential oils [112,113]. Or in another approach, improved bioformulations containing living bacteria and concentrated culture supernatants with antimicrobial metabolites have also been reported [114]. Moreover, BCA performance can be improved by genetic alterations to enhance the efficacy of selected strains for biological control. This may be achieved by conventional approaches as well as through recombinant DNA techniques. However, regulation restrictions to apply and release genetically modified organisms (GMO) into the environment must be considered since genetic manipulation is an impediment for registration of a GM-biological control agent. Genetic engineered bacteria for development of improved bioformulations may offer a good opportunity for future. This approach may include engineered strains without foreign genes but containing useful mutations in genes affecting the biocontrol or strains containing genes from other bacteria. There are several examples of genetic improvement, such as the overproduction of the antimicrobial polyketides, pyoluteorin and 2,4-diacetylphloroglucinol, in *P. fluorescens* CHA0 [115] or the enhancement of mycosubtilin production in *B. subtilis* ATCC 6633 [116].

In conclusion, in recent years there have been important advances in the knowledge of BCA for the development of commercial products for bacterial and fungal disease management. However, large-scale implementation of biological control is hampered by the limitation of commercially available and efficient BCA. Future trends should include the identification of novel BCA and require rapid and robust screening methods suitable to evaluate high numbers of candidates. Moreover, a deep study of model BCA using comparative genome analysis, and genome, transcriptome and proteome analysis will provide a valuable framework allowing for a detailed analysis of the biological mechanisms of BCA and to design strategies enhancing its beneficial action. In addition, this multi-omics approach will allow to analyze the impact of field application of bacteria on the indigenous microbiome of plants. This study would allow analyzing the environmental impact of BCA, to ensure its biosafety, and understand how to modulate the microbiome to improve the efficacy of biocontrol.

## Figures and Tables

**Table 1:** Selected bacterial biocontrol agents ^1^ of plant diseases.

Microorganism and Strain	Target Pathogen or Disease ^2^	In Vivo/In Planta Trials	Disease Reduction (%)/Application Dose/(CFU mL^−1^)	Mechanism Involved/Trait ^3^	Reference
*B. amyloliquefaciens* PPCB004	Ac, B, Cg, Fa, Lt, Pc, Pp	orange fruits	20–70/10^8^	Ab-fengycin, iturin A, surfactin	[16]
*B. amyloliquefaciens* CPA-8	Bc, Mf, Ml	cherry fruits	24–62/10^7^	Ab-fengycin-like, VOCs	[17]
*Bacillus subtilis* UMAF6614 and UMAF6639	Pf	detached melon leaves	67–74/10^8^	Ab-bacillomycin, fengycin, iturin A	[18]
*Bacillus velezensis* A17	Ea, Ps, Xa	-	-	Ab-bacillomycin, fengycin, iturin, surfactin,	[19,20]
*Lactobacillus plantarum* TC92, PM411	Ea, Psk, Xf	pear, kiwi, and strawberry plants	45–75/10^8^	CE	[21,22]
*Leuconostoc mesenteroides* CM160	BFV	-	-	Ab-mesentericin	[23]
*Pantoea agglomerans* EPS125	PF	apricot, peach, and nectarine fruits	49–61/10^7^	CE	[24,25]
*P. agglomerans* CPA-2	PF	pear fruits	50–95/10^7^	CE	[26]
*Pseudomonas chlororaphis* PCL1606	Rn	avocado plants	40/10^9^	Ab-2-hexyl, 5-propyl resorcinol	[27]
*Pseudomonas fluorescens* MVW1-2, MVP 1-4	Fop, Gt	-	-	Ab-phloroglucinol (DAPG)	[28]
*P.fluorescens* EPS62e	Ea	detached flowers, and pear plants	31–98/10^8^	CE, NC	[29]
*P. fluorescens* EPS817, EPS894	Pc	strawberry plants	76–80/10^8^	Ab-phenazines (PCA)	[30]
*Pseudomonas simiae* PICF7	Vd	olive plants	20–28/10^8^	CE/IR-local and systemic defenses	[31,32]
*Pseudomonas pseudoalcaligenes*AVO110	Rn	-	-	CE	[33]
*Streptomyces strains* CBQ-EA-2, CBQ-B-8	Mp, Rs	bean plants	60–75/10^8^	Extracellular enzyme activities	[34]
*Streptomyces sp.* VV/E1, VV/R1, VV/R4	GTD	grapevine plants	25–35/10^7^	-	[35]
*Weissella cibaria* TM128	PBF	apple fruits	50/10^8^	Ab-organic acids	[36]

^1^ Only examples of studies performed by Spanish groups are selected. ^2^ BFV, bioprotection of fresh fruits and vegetables; GTD, grapevine trunk diseases; PBF, phytopathogenic bacteria and fungi; PF, postharvest fungi; Ac, *Alternaria citri*; B, *Botryosphaeria* sp.; Bc, *Botrytis cinerea*; Cg, *Colletotrichum gloesporioides*; Ea, *Erwinia amylovora*; Fa, *Fusicoccum aromaticum*; Fop, *Fusarium oxysporum* f. sp. pisi; Gt, *Gaeumannomyces tritici;* Lt, *Lasidiplodia theobromae*; Mp, *Macrophomina phaseolina*; Mf, *Monilia fructicola*; Ml, *Monilia laxa*; Pc, *Penicillium crustosum*; Pp, *Phomopsis perse*; Pc, *Phytophthora cactorum*; Pf, *Podosphaera fusca*; Ps, *Pseudomonas syringae*; Psk, *Pseudomonas syringae* pv kiwi; Rn, *Rosellinia necatrix*; Rs, *Rhizoctonia solani*; Vd, *Verticillium dahliae*; Xa, *Xanthomonas arboricola*; Xf, *Xanthomonas fragariae*. ^3^ Ab, antibiosis; CE, competitive exclussion; IR, induced resistance; NC, nutrient competition.

**Table 2:** Some strategies for the physiological improvement of bacterial biocontrol agents.

Microorganism and Strain	Approach for the Improvement	Effect Observed on B-BCA	Reference
*Lactobacillus plantarum* PM411	Combined hyperosmotic and acid stress adaptation	Increased survival on plant surfaces and overexpression of stress-related genes.	[99]
*L. plantarum* TC92 and PM411	Mixed bacteria combined with lactic acid	Improvement of efficiency and reliability of biocontrol of fire blight.	[100]
*Pantoea agglomerans* EPS125	Combined saline osmotic stress and osmolyte amendment	Intracellular accumulation of trehalose and glycine betaine and higher tolerance to desiccation.	[101]
*Pseudomonas fluorescens* EPS62e	Combined saline osmotic stress and osmolyte amendment	Intracellular accumulation of trehalose, glucosyl-glycerol, and N-acetylglutaminylglutamine amide and improvement of cell survival on plant surfaces and after formulation.	[102,103]
*P. fluorescens* EPS62e	Nutritional enhancement combined with osmoadaptation	Improvement of fitness in plant surfaces and efficacy in biocontrol of fire blight.	[104]
*P. fluorescens* EPS817 and EPS894	Mixed bacteria producing different bioactive metabolites	Improvement of efficiency and reliability of biocontrol of *Phytophthora* root.	[30]

## Data Availability

Not applicable.
